# HOTAIR/miR‐125 axis‐mediated Hexokinase 2 expression promotes chemoresistance in human glioblastoma

**DOI:** 10.1111/jcmm.15233

**Published:** 2020-04-12

**Authors:** Jinnan Zhang, Guangyong Chen, Yufei Gao, Huaxin Liang

**Affiliations:** ^1^ Department of Neurosurgery The Third Hospital of Jilin University Changchun China; ^2^ Jilin Provincial Key Laboratory of Neuro‐oncology Changchun China

**Keywords:** chemoresistance, glioblastoma, Hexokinase 2, miR‐125

## Abstract

Drug resistance is one of the major obstacles in glioblastoma (GBM) treatments using temozolomide (TMZ) based conventional chemotherapy. Recent studies revealed that Hexokinase 2 (HK2)‐mediated glycolysis is one of the sources, as the association of chemoresistance and the expression of HK2 was confirmed in multiple cancers. However, there has been little knowledge of the functional contribution of HK2 to TMZ resistance in GBM. In our study, we found that HK2 expression is crucial for GBM proliferation and chemoresistance. In contrast to the healthy brain, HK2 expression is much higher in human GBM, especially in those patients with GBM recurrence. High HK2 expression is negatively related to the overall survival in GBM patients. HK2 depletion in GBM cells suppressed the GBM cell proliferation and increased sensitivity to TMZ‐induced apoptosis. Both HK2‐mediated glycolysis and mitochondria permeability transition pore opening (MPTP) were associated with its function in chemoresistance. Furthermore, we also revealed that the abnormal expression of HK2 was modulated by the expression of HOTAIR, a long non‐coding RNA (lncRNA). The absence of HOTAIR in GBM cells suppressed the HK2 expression in protein and mRNA level and, therefore, inhibited the cell proliferation and enhanced the cytotoxicity of TMZ both in vivo and in vitro. HOTAIR promoted the expression of HK2 by targeting mir‐125, which suppressed the GBM cell proliferation and increased the TMZ‐induced apoptosis. These findings shed light on a new therapeutic strategy in modulating HOTAIR/miR‐125, which may interfere with the expression of HK2, and enhance the therapeutic sensitivity of GBM to TMZ.

## 1 INTRODUCTION

Glioblastoma multiforme, as one of the most lethal cancers, is the primary gliomas and the most common malignant central nervous system tumour in adult patients.^1^ Currently, general treatment approaches for GBM are radiation or alkylating agent‐based chemotherapy. The most widely used alkylating agent‐based chemotherapeutics include 1,3‐bis(2‐chloroethyl)‐1‐nitrosourea (BCNU) and temozolomide (TMZ), due to their efficiency in crossing the blood‐brain barrier (BBB).^2^, ^3^ However, a subset of glioblastoma patients developed drug resistance after several months' treatment, which eventually leads to tumour recurrence.

Several studies have found elevated aerobic glycolysis in cancer cells, which was essential for maintaining chemoresistance.^4^, ^5^, ^6^ In malignant cells, aerobic glycolysis is the dominant pattern of glucose metabolization, which is known as the Warburg effect. Cells were presented with increased lactate and glycolysis production.^7^ To maintain the high glycolytic phenotype, some of the crucial molecules in glucose metabolism were deregulated. Hexokinases (HKs), for instance, are an essential enzyme in the glycolytic pathway, which catalyses the glucose into glucose‐6‐phosphate.^8^, ^9^ Hexokinase 2 (HK2) is the predominant isozyme among the four known hexokinase isoforms in human.^10^ The overexpression of HK2 was found to be associated with aerobic glycolysis in multiple tumours. HK2 was well documented to play an essential part in the Warburg effect and to be a metabolic target in cancer treatment.^11^ The increase in mitochondrial‐bound HK2 exhibited cancer‐promoting effects by inducing glycolysis and the inhibition against apoptosis.^12^ In terms of GBM, HK2 was also reported to mediate the glycolysis and suppressed the sensitivity to radiation and temozolomide‐based chemotherapy.^13^ In respect of the therapeutic potentials of HK2, understanding the mechanism and directly and selectively targeting the HK2 may provide a feasible strategy in GBM treatment.

LncRNAs, as 200 nt to ∼100 kilobases (kb) mRNA‐like transcripts, have no protein coding potential but play an essential role in gene transcription and translation, genetic and epigenetic regulation.^14^ Their dysregulation has been found in various types of carcinomas.^14^, ^15^, ^16^, ^17^ The expression of HK2 was reported to be mediated by several lncRNAs, such as HOTAIR,^18^ MALAT1,^19^ UCA1^16^ and PVT1.^17^ However, the dysregulation of HK2 in GBM remains unclear.

In our study, we showed that the abnormal expression of HK2 in GBM contributes the tumour cells proliferation and resistance to TMZ‐induced apoptosis. Importantly, the expression of HK2 is mediated by HOTAIR/miR‐125 axis. Targeting HOTAIR/miR‐125 pathway efficiently suppressed the expression of HK2 and sensitized the GBM cells to TMZ‐induced cell death, which provides a new approach against drug resistance of GBM.

## 2 MATERIAL AND METHODS

### 2.1 Clinical samples

The GBM samples used in this study were collected from 38 patients in The Third Hospital of Jilin University. These 38 GBM patients have all received TMZ chemotherapy, with 14 patients having GBM recurrence. The collection and use of these clinical specimens for this experimental study were reviewed and approved by the Ethics Committee of Jilin University, and written consents were signed by each patient. The immunochemistry staining of HK2 (Cell Signaling) in GBM samples was conducted as previously described.^20^

### 2.2 Cell culture and reagents

The U‐87 and A172 cell lines were procured from ATCC. The TMZ resistant U‐87 and A172 cells were generated as described in our previous study.^21^ The cell lines were cultured at 37°C with 5% carbon dioxide. DMEM/F12 medium (Invitrogen) supplied with 10% foetal bovine serum (FBS; HyClone). The TMZ and lactate were purchased from Sigma‐Aldrich. The TMZ powder was dissolved in sterile PBS according to the manufacturer's protocol and diluted to the desired concentration.

### 2.3 Cell death and proliferation analysis

The apoptosis of the GBM cells was analysed by Hoechst 33258 staining (Invitrogen) as previously described.^22^ Alternatively, the apoptotic cells were also analysed by annexin‐V/PI staining (Thermo Fisher) followed by flow cytometry (BD accuri C6, BD Biosciences) analysis as previously described.^22^

The cell viability was determined by MTT assay as described in our previous study.^21^ Proliferation was analysed using BrdU incorporation assay (Promega) in accordance with the manufacturer's protocol. Cells were normalized by a standard curve.

### 2.4 Colony formation assay

Cells were seeded into 6‐well plates (1.5 × 10^3^ cells per well) to reach adhesion and then treated with TMZ for 24 hours. Then, the medium was discarded for adding fresh medium into each well. The cells were then incubated for another 12 days. The colonies were visualized by staining with crystal violet buffer (0.1% in 1% formaldehyde).

### 2.5 Quantitative real‐time PCR (qRT‐PCR) assay

Total RNA from GBM cells or frozen tissue samples was extracted by the RNEasy kit (QIAGEN), and the cDNA was synthesized with the QuantiTect RT kit (QIAGEN). SYGR green (Invitrogen) and the Chromo4 Real‐Time PCR detector (Bio‐Rad Laboratories) were used to perform the qRT‐PCR assay. The primers were as follows: HK2, 5′‐CAAAGTGACAGTGGGTGTGG‐3′, and 5′‐GCCAGGTCCTTCACTGTCTC‐3′; HOTAIR: 5′‐CAGTGGGGAACTCTGACTCG‐3′, and 5′‐GTGCCTGGTGCTCTCTTACC‐3′; Glyceraldehyde 3‐phosphate dehydrogenase (GAPDH): 5′‐GTCAACGGATTTGGTCTGTATT‐3′, and 5′‐AGTCTTCTGGGTGGCAGTGAT‐3′.

### 2.6 Western blot

The expression of proteins was analysed by Western blot as previously described.^21^ Antibodies including those for cleaved caspase‐3, HK2, cytochrome c, tubulin, Ki‐67 and actin were purchased from Cell Signaling. The mouse and rabbit source of second antibodies were purchased from Santa Cruz and diluted with 1:2000 for use. The expression of Ki‐67 was used as a marker of cell proliferation. The expression of cleaved caspase‐3 and cytochrome c release in the cytosol was used as markers for apoptosis.

### 2.7 Lentivirus shRNA transfection

Target sequences were inserted into a pLKO.1 plasmid (Addgene) for the construction of sh‐HOTAIR and sh‐HK2. The shRNA sequences are as follows: sh‐HOTAIR, 5′‐GAGACACATGGGTAACCTA‐3′; and sh‐HK2, 5′‐GGTTGACCAGTATCTCTAC‐3′. Lentiviral vectors with packaging vectors pMDLg/pRRE, pMD2G and pRSV‐REV (Addgene) were cotransfected into HEK293T cells using Lipofectamine 2000. The cells were lentivirus infected and selected with puromycin (2 μg/mL) for 72 hours to establish stable cell lines.

### 2.8 Transfections of DNA plasmids

Lipofectamine 2000 (Invitrogen) was used for the transfection in accordance with the manufacturer's instructions. All the siRNA for VDAC (ACACUAGGCACCGAGAUUA), miR‐125 mimic, miR‐125 antagonist and controls were synthesized and purchased from GenePharma.^23^

### 2.9 RNA pull‐down assay

The RNA immunoprecipitation was performed by using Dynabeads™ MyOne™ Streptavidin C1 kit (Thermo Fisher) in accordance with manufacturer's instructions. About 1 × 10^7^ U87 in total were harvested, lysed in the RIPA lysis buffer. The biotin‐labelled miR‐125 and control oligo probe was incubated with C‐1 magnetic beads at 25°C for 1 hour to generate probe‐coated beads as previously described.^24^ Cell lysate with miR‐125 or control probe was incubated at 4°C overnight, followed by washing with wash buffer. The purified RNA was subjected extraction using RNeasy Mini Kit (QIAGEN), followed by qRT‐PCR analysis using the primer of HOTAIR.

### 2.10 Metabolic assays

Lactate assay was performed and normalized to cell number following the manufacturer's instructions (Eton Bioscience). The YSI 2700 biochemistry analyzer (YSI) was used to measure the concentration of glucose. The ATP level was analysed by CellTiter‐Glo 2.0 (Thermo Fisher) as described by the manufacturer's instruction.

### 2.11 Animal studies

The animal experiments were performed according to the Animal Ethics guidelines of Jilin University, and the studies were approved by the Institutional Animal Ethics Committee (IEC) of Jilin University. Severely immunocompromised female nude mice were used in the in vivo tumorigenicity experiments. For the subcutaneous tumour model, 1 × 10^6^ of parental U87 cells with control or HOTAIR shRNA transfection were subcutaneously injected into 6‐week‐old nude mice (n = 5). For orthotopic xenograft model, 1 × 10^5^ of parental U87 cells with control or HOTAIR shRNA transfection were injected into the brains of nude mice. Seven days after the tumour cell implanting, the mice were given TMZ (i.p. 25 mg/kg, three times per week) or PBS (Control) in relative groups. The tumours were measured every 3 days, and the volume of the tumour was calculated using the formula: 4/3*π* (√major axis/2 × minor axis/2). After mice killing, tumours were dissected and fixed and embedded in paraffin for TUNEL staining or stored at −80°C for Western blot analysis.

### 2.12 Statistical analysis

The data were expressed as mean ± SEM for continuous variables and frequencies (%) for categorical variables. Students' *t* test or one‐way ANOVA were used in comparison with the data in different groups. *P* < .05 was considered statistically significant. All the experiments were repeated in triplicate.

## 3 RESULTS

### 3.1 Higher HK2 expression is correlated with chemoresistance in GBM

**FIGURE 1:**
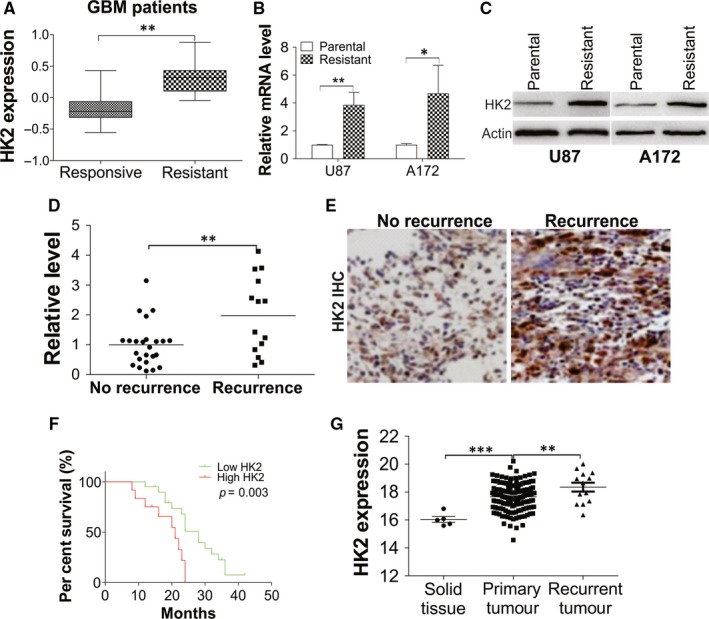
Dysregulation of HK2 in TMZ resistant GBM leads to poor survival. A, The expression of HK2 from GEO database (GSE2221). B, The HK2 mRNA level in U87 and A172 parental and TMZ resistant cells. C, Western blot of HK2 in U87 and A172 parental and TMZ resistant cells. D, The mRNA level of HK2 in GBM tissues from 38 patients received TMZ treatment (responsive patients: 24 patients without tumour recurrence; resistant patients: 14 patients with tumour recurrence). E, The representative IHC of HK2 in tumours from TMZ responsive and resistant GBM patients. F, The overall survival of 38 GBM patients with different level of HK2 expression. G, The HK2 expression from TCGA GBM RNAseq (IlluminaHiSeq) dataset (N = 671) (https://xenabrowser.net/heatmap/). Data were represented in means ± SEM. *P < .05; **P < .01; ***P < .001

To elaborate the role of HK2 expression in GBM chemoresistance, we analysed the expression of HK2 in GESA database (GSE2221) by dividing the 30 samples into two groups, the chemotherapy drugs responsive and resistant groups. We found that HK2 expression is higher in the chemoresistant group (Figure 1A, *P* = .0024). Consistently, the HK2 expression was also higher in our previously developed TMZ resistant U87 and A172 cells^21^ in both mRNA and protein levels (Figure 1B,C). To study the relationship between HK2 expression and chemotherapy response in GBM patients, we further analysed the HK2 expression in tissue samples obtained from 38 GBM patients with TMZ treatment. Among these 38 patients, 14 patients had GBM recurrence, which were grouped as TMZ resistant patients. We then further compared the HK2 expression level in the tumour from these 38 patients with or without GBM recurrence. We found that patients with tumour recurrence had higher HK2 expression (Figure 1D,E). GBM patients with higher HK2 expression also had poorer survival outcomes (Figure 1F). Consistently, the data from the cancer browser (https://xena.ucsc.edu/welcome-to-ucsc-xena/) also suggested that HK2 mRNA expression level is higher in the primary GBM tumours, when compared with the solid normal tissues (Figure 1F). Moreover, the HK2 expression level was much higher in the recurrent GBM tumours (Figure 1F). Therefore, our data supported that HK2 expression is higher in GBM tumours, especially in chemoresistant GBM patients.

### 3.2 Depletion of HK2 enhanced the cytotoxicity of TMZ in GBM cell lines

**FIGURE 2:**
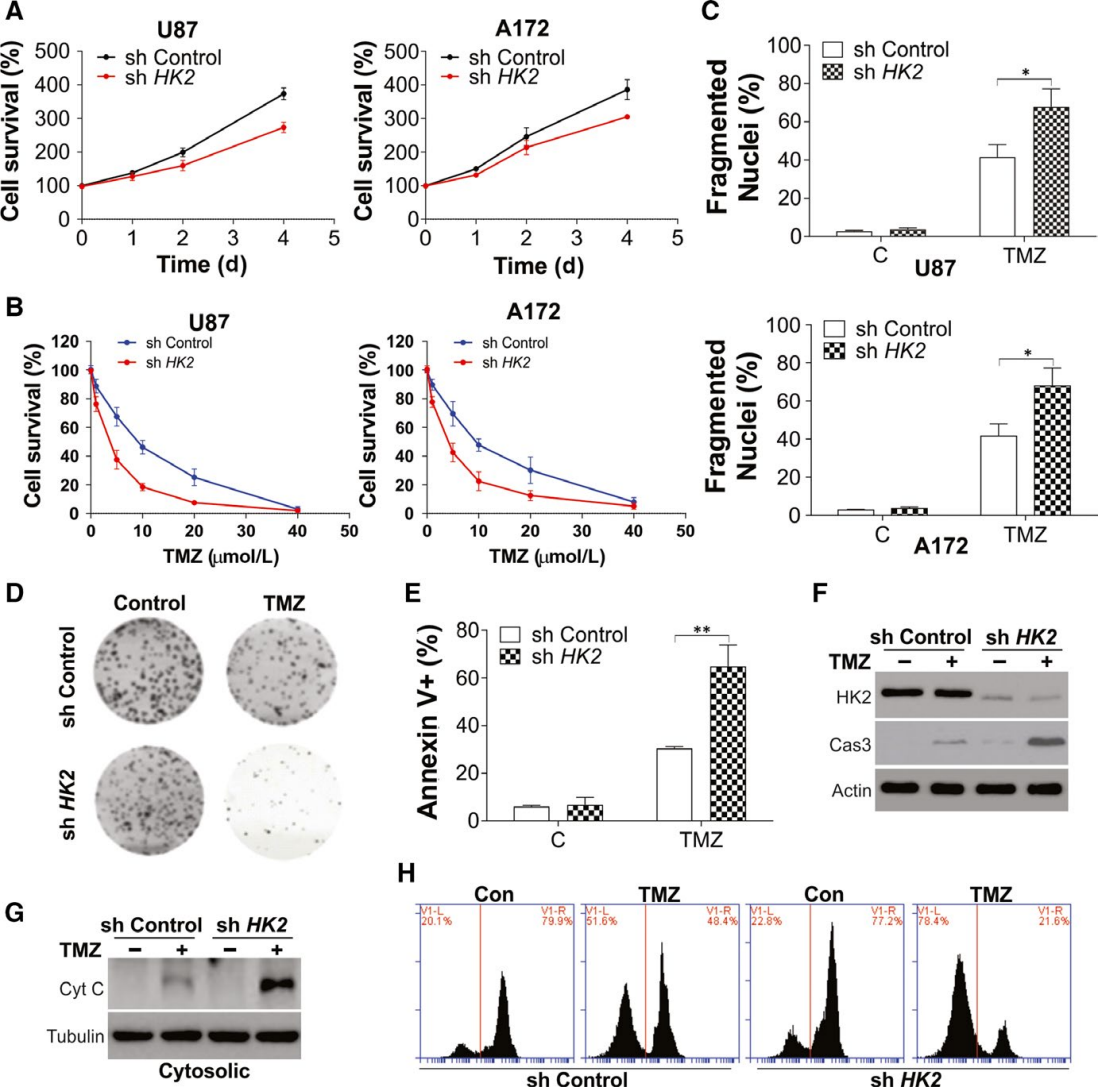
HK2 expression mediated GBM cell proliferation and chemosensitivity. A, The growth of U87 (left) and A172 (right) with stable transfection of control or HK2 shRNA by lentivirus. B, The cell viability of U87 (left) and A172 (right) with stable transfection of control or HK2 shRNA upon different dosages of TMZ treatment for 24 h. C, The apoptosis of U87 (upper) and A172 (lower) with stable transfection of control or HK2 shRNA upon 10 µmol/L TMZ treatment for 24 h. D, The colony formation of U87 cells treated as in (C). E, The annexin‐V/PI staining of U87 cells treated as in (C) was analysed by flow cytometry. F, The expression of HK2 and cleaved caspase‐3 (cas‐3) in U87 cells treated as in (C). G, The cytochrome c in cytosolic fraction of U87 cells treated as in (C). H, The flow cytometry analysis of U87 cells treated as in (C) followed by mitotracker‐red staining. Data were represented in means ± SEM. *P < .05; **P < .01

Since our data suggested that high HK2 expression might contribute to GBM chemoresistance, we further studied the influence of HK2 depletion on the proliferation and response to TMZ treatment in GBM cells. The depletion of HK2 in U87 and A172 cell lines by lentivirus shRNA transfection significantly inhibited cell growth compared with the control shRNA transfected cells (Figure 2A). The proliferation of GBM cells was also compromised by HK2 knockdown confirmed by the Brdu assay (Figure S1A). When treated with TMZ, HK2 deficient U87 and A172 cells were more sensitive to different dosages of TMZ (Figure 2B) and had lower LC50 values (Figure S1B). As apoptosis is one of the major cell death induced by TMZ, we then analysed the apoptotic cell death induced by TMZ using Hoechst‐33258 nuclear staining. The absence of HK2 did not induce apoptosis but enhanced the apoptosis in TMZ treated U87 and A172 cells (Figure 2C). The crystal violet staining also revealed that the absence of HK2 reduced the cell viability, as well as enhanced the cytotoxicity of TMZ (Figure 2D). The Annexin‐V/PI staining analysed further confirmed that HK2 depletion did not induce apoptosis but sensitized the U87 cells to TMZ‐induced apoptosis (Figure 2E). HK2 absence in U87 cells also enhanced the cleavage of caspase‐3 upon TMZ treatment, suggesting higher apoptosis (Figure 2F).

It has been previously reported that HK2 could translocate to the outer mitochondrial membrane and regulate the interaction with members of the permeability transition pore.^25^ This progress could control the cytochrome c release, which was essential in the intrinsic apoptotic pathways.^25^ In our study, we also found that depletion of HK2 enhanced the release of mitochondrial cytochrome c (Figure 2G), as well as the loss of mitochondrial potential (Figure 2H) upon TMZ treatment in U87 cells. Therefore, these results indicated that HK2 expression mediated the chemoresistance in GBM by suppressing the TMZ‐induced apoptosis.

### 3.3 HK2‐mediated lactate production also contributes to GBM chemoresistance

**FIGURE 3:**
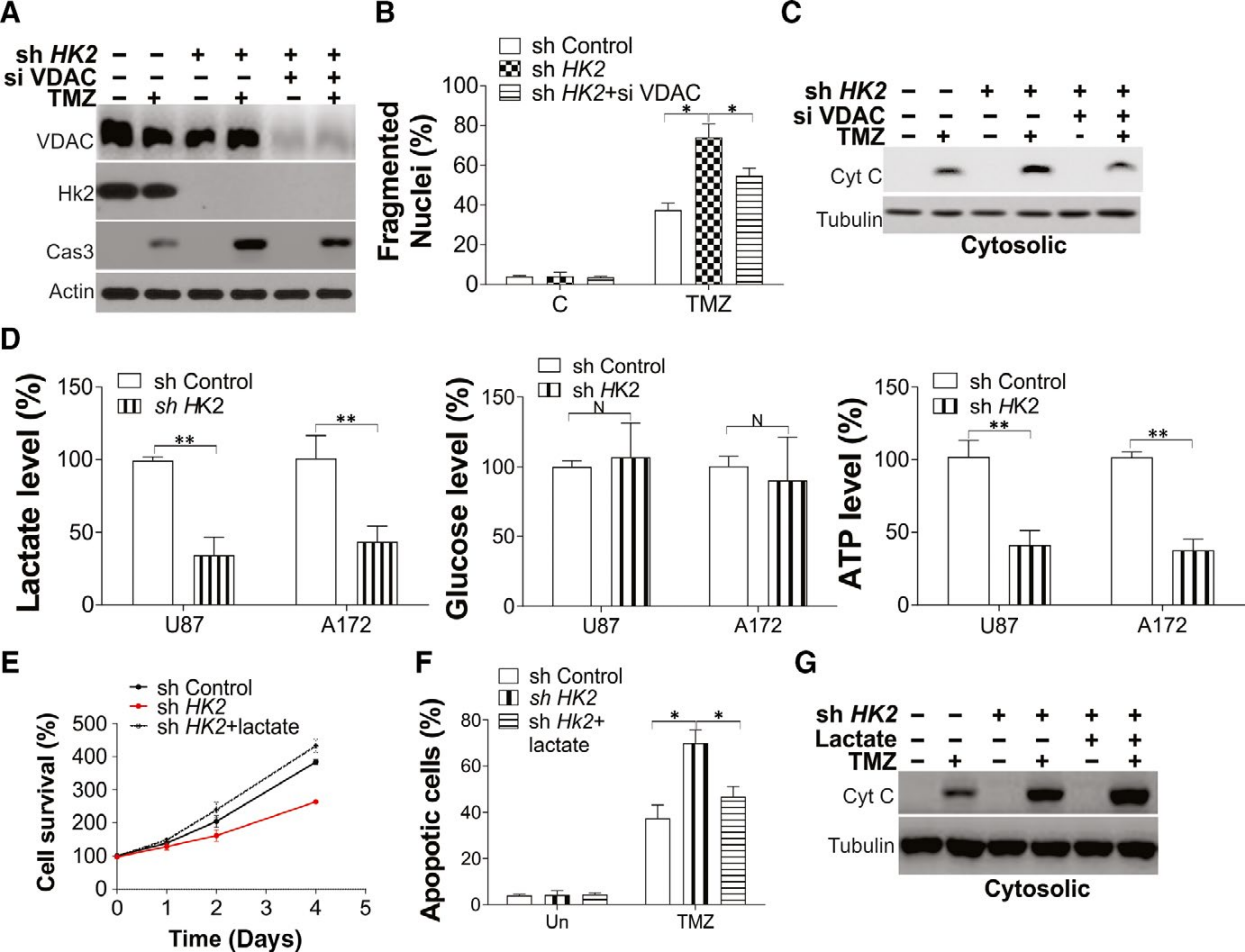
HK2 mediated mitochondria opening and glycolysis contributes to chemoresistance in GBM. A‐C, U87 cells stably transfected with control or HK2 shRNA were transfected with VDAC siRNA for 12 h and treated with 10 µmol/L TMZ for another 24 h. A, The expression of indicated proteins in total cell lysates. B, The apoptosis in each group of cells. C, The cytochrome c expression in cytosolic fractionation. D, The lactate, glucose and ATP level in U87 and A172 cells stably transfected with control or HK2 shRNA. E, F, The U87 cells stably transfected with control or HK2 shRNA were treated with 40 mM lactate. E, The growth of cells in each group. F, The apoptosis of cells in each group treated with 10 µmol/L TMZ for 24 h. G, The cytochrome c expression in cytosolic fractionation upon TMZ treatment. Data were represented in means ± SEM. ^N^ *P* > .05; *P < .05; **P < .01

Hexokinase 2 was reported to translocate to mitochondria by binding with VDAC and mediate the mitochondria permeability transition pore (MPTP) opening. We then investigated whether HK2 suppressed the TMZ‐induced apoptosis via VDAC binding. The depletion of VDAC by siRNA partially reduced the cleavage of caspase‐3 in HK2 stable knockdown U87 cells in response to TMZ treatment (Figure 3A). Consistently, the Hoechst‐33258 staining indicated that the absence of VDAC could not wholly suppress TMZ‐induced apoptosis in HK2 depletion U87 cells (Figure 3B), although it suppressed the mitochondria cytochrome c release (Figure 3C). Therefore, these results suggested that HK2 suppression of MPTP opening only partially contributes to the chemoresistance in GBM.

Since HK2 is an vital regulator of glycolysis, which has been previously reported to promote cancer progression,^26^, ^27^ we suggested that HK2‐driven glycolysis was also involved during the process of the chemoresistance of GBM. Firstly, we measured several glycolysis productions in the HK2 stable knockdown cell lines, including lactate, glucose and ATP. To ensure that the metabolic production changes resulted from the shift of HK2 levels but not a result of the proliferation rate change, we normalized the value of these product assay to the growth rate of cells as analysed by MTT assay. Consistent with the previous study,^28^ we found knockdown of HK2 suppressed the lactate and ATP production in U87 and A172 cells (Figure 3D). However, HK2 depletion did not affect glucose uptake (Figure 3D). The U87 and A172 TMZ resistant cells, which had higher HK2 expression, also showed increased lactate and ATP level, when compared with parental cells (Figure S2A). To investigate whether glycolysis changes by HK2 contribute to the chemoresistance in GBM, we cultured the U87 cells by supplement of lactate. The supplement of lactate recovered the cell proliferation, which was suppressed by HK2 depletion (Figure 3E, Figure S2B), which phenocopied the HK2 depletion. In terms of apoptosis, supplement of lactate also partially suppressed the TMZ‐induced apoptosis in HK2 deficient U87 cells, as indicated by Hoechst‐33258 nuclear staining (Figure 3F). However, the lactate supplement did not change the TMZ‐induced cytochrome c release, which was enhanced by HK2 depletion (Figure 3G). Therefore, our results suggested that both HK2‐mediated MPTP opening and glycolysis changes are necessary for its function in GBM chemoresistance.

### 3.4 Dysregulation of HOTAIR promotes higher HK2 expression in chemoresistant GBM cells

**FIGURE 4:**
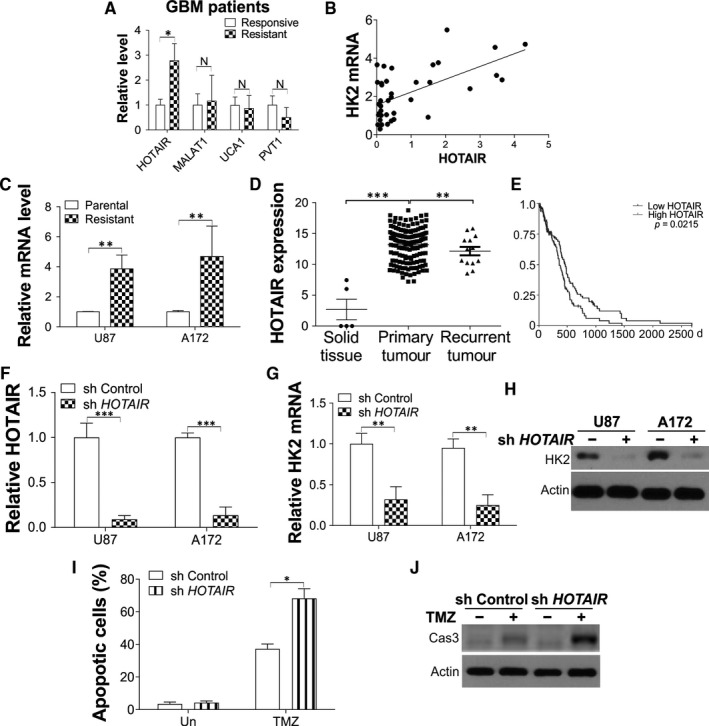
The dysregulation of HK2 is mediated by HOTAIR in TMZ resistant GBM cells. A, The expression of indicated lncRNA in TMZ responsive or resistant GBM patients. B, The expression correlation between HOTAIR and HK2 in 38 GBM patients. *R* = .368. C, The mRNA level of HOTAIR in U87 and A172 parental or TMZ resistant cells. D, The HOTAIR expression from TCGA GBM RNAseq (IlluminaHiSeq) dataset (N = 671) (https://xenabrowser.net/heatmap/). E, Kaplan‐Meier curves for comparing overall survival (OS) of GBM patients with high and low expression levels of HOTAIR. F, The expression of HOTAIR in U87 and A172 cells transfected with control or HOTAIR shRNA lentivirus. G, H, The mRNA (G) and protein (H) level of HK2 in U87 and A172 cells transfected with control or HOTAIR shRNA lentivirus. I, The apoptosis of U87 with stable transfection of control or HK2 shRNA upon 10 µmol/L TMZ treatment for 24 h. J, The cytochrome c expression in cytosolic fractionation of U87 cells treated as in (I). Data were represented in means ± SEM. ^N^ *P* > .05; *P < .05; **P < .01, ***P < .001

We further studied the mechanism of high HK2 expression in chemoresistant GBM. The long non‐coding RNA (lncRNA) was reported as an essential regulator in GBM. We then investigated the expression of several previous reported HK2 related lncRNAs in GBM patients, including HOTAIR,^18^ MALAT1,^19^ UCA1^16^ and PVT1.^17^ Only HOTAIR expression was found up‐regulated in the TMZ resistant GBM patients and cells (Figure 4A, Figure S3A). The expression of HOTAIR was also positively correlated with the expression of HK2 in those 38 GBM patients (Figure 4B). Consistently, the expression of HOTAIR was also higher in the TMZ resistant U87 and A172 cells (Figure 4C) and further confirmed that HOTAIR was up‐regulated in chemoresistant GBM. The TGCA database analysis indicated that HOTAIR expression was also higher in GBM primary tumours (Figure 4D) and led to reduced survival of GBM patients (Figure 4E). To investigate the effect of HOTAIR expression onHK2 expression, we used the lentivirus shRNA to knockdown the endogenous HOTAIR in U87 and A172 cell lines (Figure 4F). Depletion of HOTAIR suppressed the expression of HK2 in mRNA (Figure 4G) and protein level (Figure 4H). Similar to the effects of HK2 knockdown, absence of HOTAIR inhibited the proliferation of U87 cells (Figure S3B,C) and enhanced the TMZ‐induced apoptosis in U87 cells (Figure 4I,J). Therefore, our data suggested that the dysregulation of HK2 in chemoresistant GBM is caused by the abnormal expression of HOTAIR, which also contributes to the chemoresistance in GBM.

### 3.5 HOTAIR promotes HK2 expression in GBM by targeting miR‐125

**FIGURE 5:**
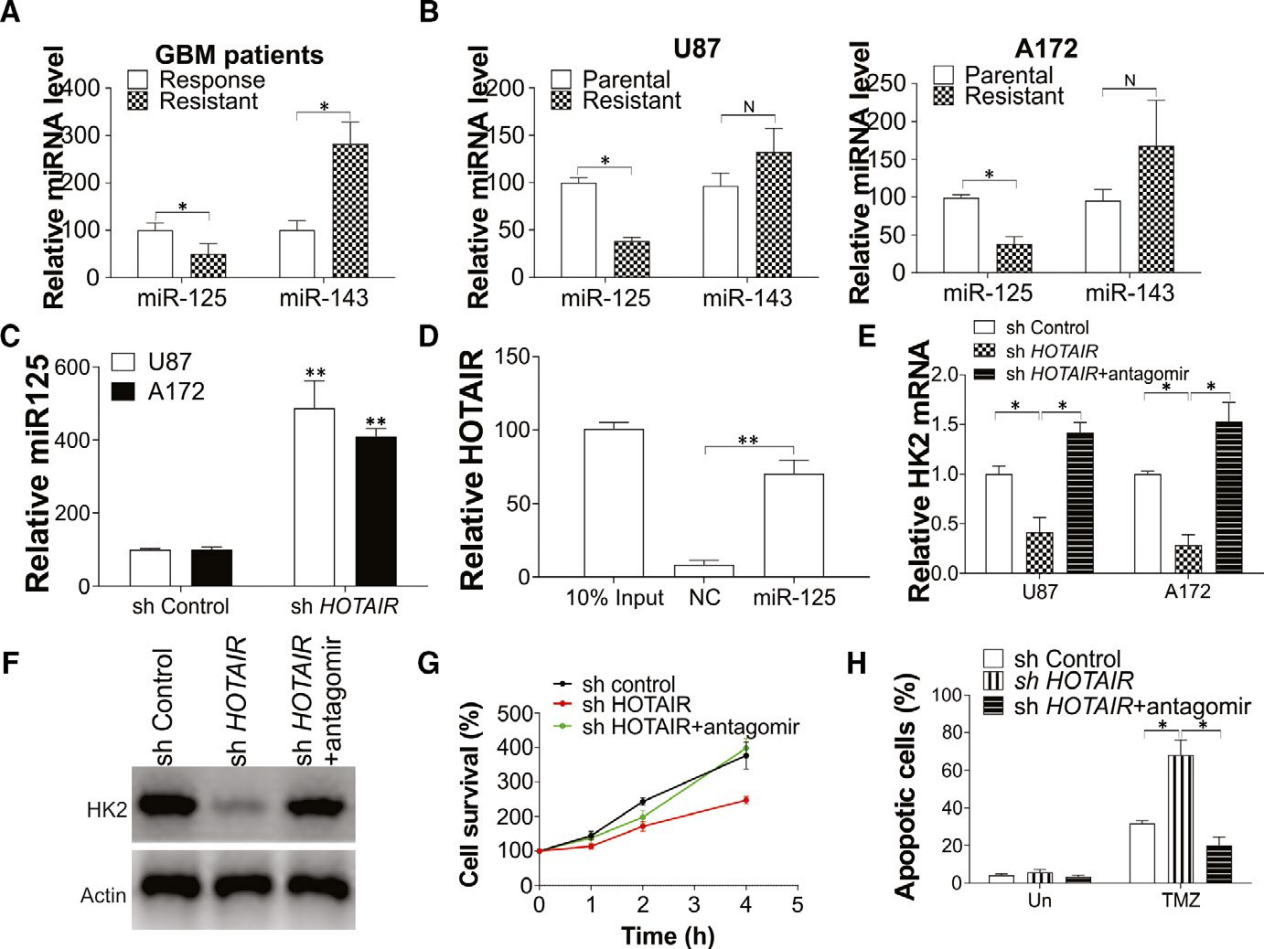
Mir‐125 is the target of HOTAIR in mediating HK2 expression. A, The expression of miR‐125 and miR‐143 in TMZ responsive or resistant GBM patients. B, The expression of miR‐125 and miR‐143 in U87 and A172 parental or TMZ resistant cells. C, The expression of miR‐125 in U87 and A172 transfected with control or HOTAIR shRNA lentivirus. D, The interaction of HOTAIR and miR‐125 interaction were analysed by biotin‐labelled miR‐125 pull‐down assay. E‐H, The U87 and A172 cells stably transfected with control or HOTAIR shRNA were transfected with miR‐125 antagomir. E, The HK2 mRNA level. F, The HK2 protein expression level. G, The cell growth of U87 cells. H, The apoptosis of U87 cells in each group treated with 10 µmol/L TMZ treatment for 24 h. Data were represented in means ± SEM. ^N^ *P* > .05; *P < .05; **P < .01

LncRNAs commonly exert their effects by interacting with downstream target miRNAs.^29^ It was reported that HOTAIR mediated the expression of HK2 by targeting miR‐125 and miR‐143.^15^ Therefore, we investigated these two miRNAs expression in chemoresistant GBM patients. Our results showed that only miR‐125 was down‐regulated when compared with chemo‐responsive patients (Figure 5A). Similarly, miR‐125 was also down‐regulated in TMZ resistant U87 and A172 cell lines (Figure 5B). The expression of miR‐125 was up‐regulated by HOTAIR knockdown in U87 and A172 cell lines (Figure 5C). A specific biotin‐labelled miR‐125 probe was used to further verify miR‐125 is the target of HOTAIR. And the probe was able to capture HOTAIR compared with the non‐specific control (NC) group (Figure 5D). Therefore, these data suggested that miR‐125 might be the target of HOTAIR in mediating chemoresistance in GBM. To further investigate whether miR‐125 is necessary for HOTAIR mediated HK2 expression, we transfected the HOTAIR stable knowdown U87 and A172 cells with miR‐125 antagomir. Consistent with our hypothesis, inhibition of miR‐125 abolished the suppressive effect of HOTAIR depletion on HK2 expression in mRNA and protein level (Figure 5E,F). Furthermore, transfection of miR‐125 antagonists in U87 cells recovered the cell proliferation (Figure 5G) and TMZ resistance (Figure 5H), which was suppressed by HOTAIR depletion. Reversely, transfection of miR‐125 mimic in U87 cells suppressed the expression of HK2 in protein and mRNA level (Figure S4A,B) and, therefore, inhibited the GBM cells proliferation (Figure S4C). Mir‐125 mimic transfection also sensitized the U87 cells to TMZ‐induced apoptosis (Figure S4D), which was similar to the effect of HOTAIR or HK2 depletion. Our results indicated that miR‐125 may be the downstream target of HOTAIR for HK2 regulation in GBM.

### 3.6 HOTAIR mediated the GBM chemoresistance in vivo

**FIGURE 6:**
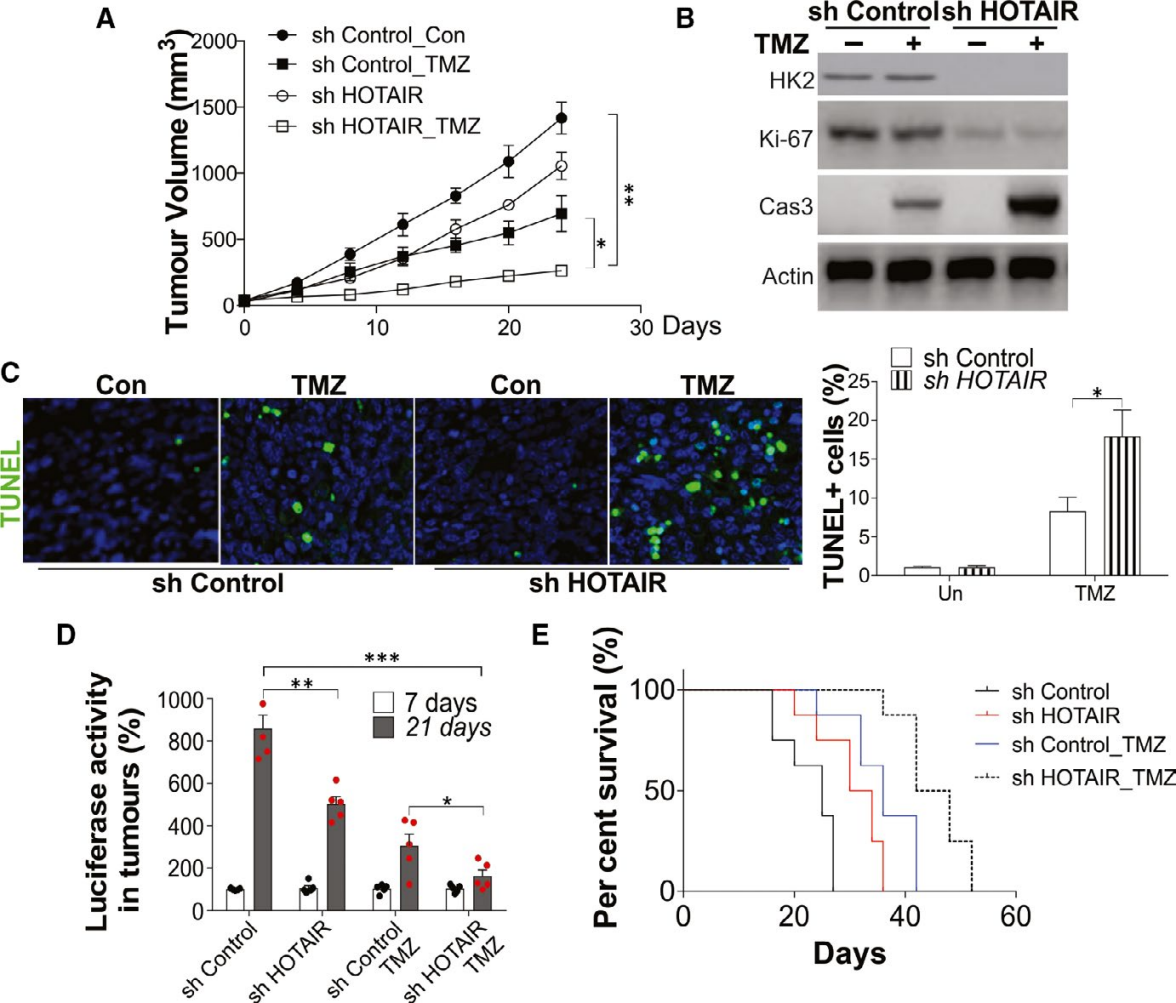
Depletion of HOTAIR enhanced the therapy effect of TMZ in vivo. A, The growth of tumours in nude mice xenografted with U87 cells stably transfected with control or HOTAIR shRNA and treated with 25 mg/kg TMZ for three times per week. B, The expression of indicated proteins in each group of tumours. C, The representative pictures of TUNEL staining in each group of tumours (left), and the statistic calculation of the percentage of TUNEL positive signals (right). D, E, U87 cells with control or HOTAIR shRNA stable transfection were intracranially injected into nude mice (1 × 10^5^ per mice, n = 5) and treated with 25 mg/kg TMZ for three times per week. The tumorigenicity was monitored. D, The luciferase activity in tumour monitored in 7 and 21 d after tumour inoculation. E, Survival analysis was calculated by Kaplan‐Meier curve. Data were represented in means ± SEM. *P < .05; **P < .01, ***P < .001

To further study the effect of HOTAIR stable knockdown on subcutaneous xenografts in vivo, U87 with control or HOTAIR shRNA stable transfection were subcutaneously transplanted into nude mice. Two weeks after tumour innovation, the mice were given TMZ (i.p. 25 mg/kg, three times per week) or PBS (Control) for 24 days. The depletion of HOTAIR suppressed the tumour growth and enhanced the suppressive effect of TMZ treatment (Figure 6A). The Western blot of tumour tissues revealed the HOTAIR knockdown suppressed the expression of HK2 and Ki‐67, a marker of proliferation (Figure 6B). Absence of HOTAIR in the tumour also enhanced the expression of cleavage caspase‐3 (Figure 6B) and increased the TUNEL positive cells in tumour tissue upon TMZ treatment (Figure 6C), suggesting higher apoptosis was induced.

Next, we evaluated the effect of HOTAIR silence on TMZ therapeutic efficacy on intracranial tumours. The Luc reporter gene labelled U87 cells were used to establish the model intracranial tumour in vivo. Nine days after the implantation of tumour cells, mice with intracranial U87 reporter cells were administered with TMZ or PBS. Consistent with the subcutaneous tumour model, depletion of HOTAIR reduced the tumour volume in intracranial tumours (Figure 6D) and extended the survival of mice (Figure 6E). Moreover, TMZ treatment also achieved better therapy effect on the mice with HOTAIR depletion tumours, which had smaller tumours and better survival (Figure 6D,E). Collectively, these results indicated that HOTAIR mediated the expression of HK2, which contributes to tumour growth and chemosensitivity in vivo.

## 4 DISCUSSION

Most cancer cells achieve their anabolic requirements by reprogramming cellular glucose metabolism. The GBM was reported to display a comprehensive glucose metabolism reprogramming among different types of cancers.^30^ By converting glucose to glucose‐6‐phosphate (G‐6‐P), HK2 catalyses the first committed step of glucose metabolism, which then initiates major pathways of glucose utilization. Therefore, HK2 is confirmed to play an important regulating rule in glucose metabolism and as a tumour promoter in multiple cancers. As to its role in GBM, HK2 was also reported to promote GBM tumour growth by aerobic glycolysis.^13^ However, it remains unclear whether dysregulation of HK2 is correlated with the chemoresistance in GBM. In this study, we found that the GBM patients received TMZ treatment with tumour recurrence has higher HK2 expression. The TMZ resistant GBM cells also have higher HK2 expression than the parental cells, suggesting that HK2 expression is higher in the chemoresistance GBM in vivo and in vitro. Deletion of HK2 suppressed the proliferation of GBM cells, as well as sensitized the GBM cells to TMZ‐induced apoptosis. Mechanically, we found that HK2 mediated mitochondria membrane opening and lactate production contribute to GBM cell sensitivity to TMZ treatment. In terms of HK2 dysregulation, we also found that HOTAIR/miR‐125 axis was a crucial regulator of HK2 expression in chemoresistant GBM cells. These results showed that HK2 may play an essential role in the regulation of chemoresistance in GBM. And targeting the HOTAIR/miR‐125 pathway can be a potential strategy to overcome HK2 dysregulation.

Mitochondrial HK2 exhibited cancer‐promoting effects by inducing glycolysis and the inhibition against apoptosis, which make it as an attractive drug target for human cancers.^31^, ^32^ Other reports have suggested that VDAC participated in the regulation of HK2's dual oncogenic activities in survival and glycolysis.^33^, ^34^ It was well documented that the mitochondrial translocation of HK2 promotes glucose phosphorylation and leads to lactate formation, which promotes cancer cell proliferation.^12^, ^13^ It was also demonstrated in o in vitro experiments that stable depletion of HK2 inhibited aerobic glycolysis, which was exemplified by the decrease in cellular lactate and ATP levels. Other than suppressing the GBM cell proliferation, depletion of HK2 also sensitized the GBM cells to the chemotherapeutic agent, TMZ. It was previously shown that the interaction between HK2 and VDAC in the mitochondria could regulate the cytochrome c release and apoptosis, although the molecule mechanism of this progress was still not well established.^25^, ^34^ However, disrupting the interaction of mitochondria and HK or the exposure to pro‐apoptotic stimuli was proved to induce HK to dissociate from the mitochondria rapidly and ultimately induce the release of cytochrome c and apoptosis, regardless of the Bax and Bak existence,^25^ suggesting a mitochondrial permeability transition pores independent apoptotic pathway. Our data also supported that the depletion of HK2 enhanced the mitochondria cytochrome c release and potential loss by treatment of TMZ. However, inhibition of VADC did not completely abandon the chemosensitivity induced by HK2 depletion, which is also verified that other mechanisms of HK2 in mediating chemoresistance in GBM cells. Since the production of lactate was reported to suppress the apoptosis induced by chemotherapy drugs,^35^ our data supported the lactate production mediated by HK2 dependent glycolysis also contributes to the resistance of TMZ in GBM cells. Therefore, our study supported that the dual function of HK2 in glycolysis and mitochondria binding with VADC modulates the chemotherapy resistance in GBM.

Despite the potential therapeutic effect of HK2, the direct and selective HK2‐targeted strategy is not yet conceivable. Our results showed that HOTAIR up‐regulation in chemoresistant GBM leads to the induction of HK2. HOTAIR is known as a lncRNA with notable involvement in the reprogramming of chromatin organization and metastasis of cancer.^36^, ^37^ Literatures have indicated the high expression of HOTAIR in different types of cancers, including GBM.^36^, ^38^ In GBM, *HOTAIR* was involved in the invasion, proliferation, colony formation, cell cycle, tumour growth in mice and the overall survival of GBM patients.^20^, ^38^ However, the mechanism of the aberrant activation of *HOTAIR* in GBM still remains elusive. Our data revealed *HOTAIR* is overexpressed in primary GBM tumour, particularly in recurrent GBM patients. The high HOTAIR expression in chemoresistant GBM leads to high expression of HK2, which promotes glycolysis and chemoresistance. Based on our observation in vitro and in vivo that HOTAIR was dysregulated in chemoresistant GBM, the molecular mechanism underlying the regulation of HOTAIR on GBM chemoresistance was investigated. The crosstalk of lncRNA, miRNA and mRNA has been identified widely: the lncRNA functions on competing for the elements for miRNA response, thus acts as a sponge of miRNA and suppresses the binding between endogenous miRNAs and their target genes.^39^ In our present study, we identified the bond of miR‐125 and the 3′UTR of HOTAIR. Interestingly, miR‐125 was also reported to inhibit the expression of HK2 in oesophageal squamous cell carcinoma and acute myeloid leukaemia through targeting HK2.^15^, ^16^ Here, we further demonstrated that miR‐125 could down‐regulate the HK2 expression. Therefore, our results provided a novel mechanism for HK2 dysregulation in chemoresistant GBM.

In conclusion, our results indicated that HOTAIR is the upstream mediator of HK2 through sequestering miR‐125, which led to the impairment of the glycolysis balance in GBM. Both HK2 dependent glycolysis and MPTP opening were involved in the HOTAIR/miR‐125/HK2‐mediated GBM chemoresistance. Elaboration on the HOTAIR/miR‐125 and miR‐125/HK2 pathways may provide a better understanding of chemoresistance in GBM, and new targets for the prevention and treatment of GBM.

## CONFLICT OF INTEREST

The authors declare no conflict of interest.

## AUTHOR CONTRIBUTIONS

HXL and YFG designed the study. JNZ performed experiment and analysed the data. JNZ drafted and wrote the manuscript. GYC revised the manuscript. All authors read and approved the final manuscript.

## Supporting information

Fig S1‐S4

## Data Availability

The datasets used and/or analysed during the current study are available from the corresponding author on reasonable request.
