# Supplementary material for: HOTAIR/miR‐125 axis‐mediated Hexokinase 2 expression promotes chemoresistance in human glioblastoma
Source: J Cell Mol Med. 2020 Apr 12;24(10):5707–17. doi: 10.1111/jcmm.15233 (PMC7214183; doi:10.1111/jcmm.15233)
Supplement: Supplementary file 1 — Fig S1‐S4 [file JCMM-24-5707-s001.docx]

**Supplemental**


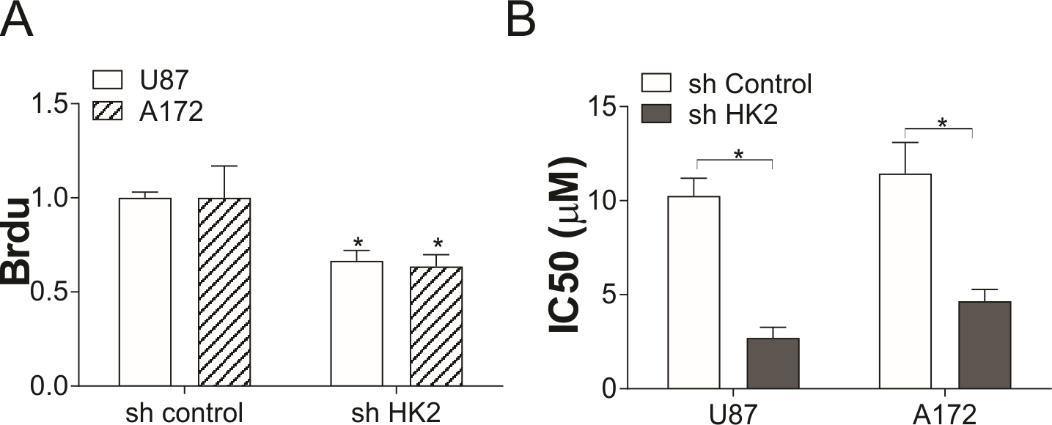


Fig. S1. Depletion of HK2 suppressed the GBM cell proliferation and chemoresistance. (A) The Brdu assay analysis of U87 and A172 cells with control or HK2 shRNA transfection. (B) The 24 h TMZ IC50 value of U87 and A172 cells with control or HK2 shRNA transfection. Data were represented in means ± SEM. *, p<0.05.


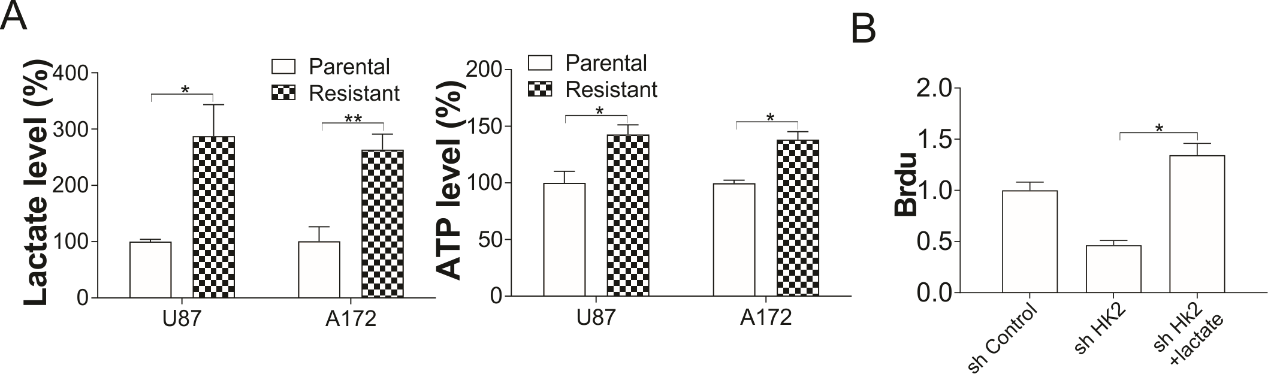


Fig. S2. The production of lactate by GBM cells enhanced the cell proliferation. (A) The production of lactate and ATP in U87 and A172 parental and TMZ resistant cells. (B) The Brdu assay of U87 and A172 cells with control or HK2 shRNA transfection supplemented with 40 mM lactate. Data were represented in means ± SEM. *, p<0.05; **, p<0.01.


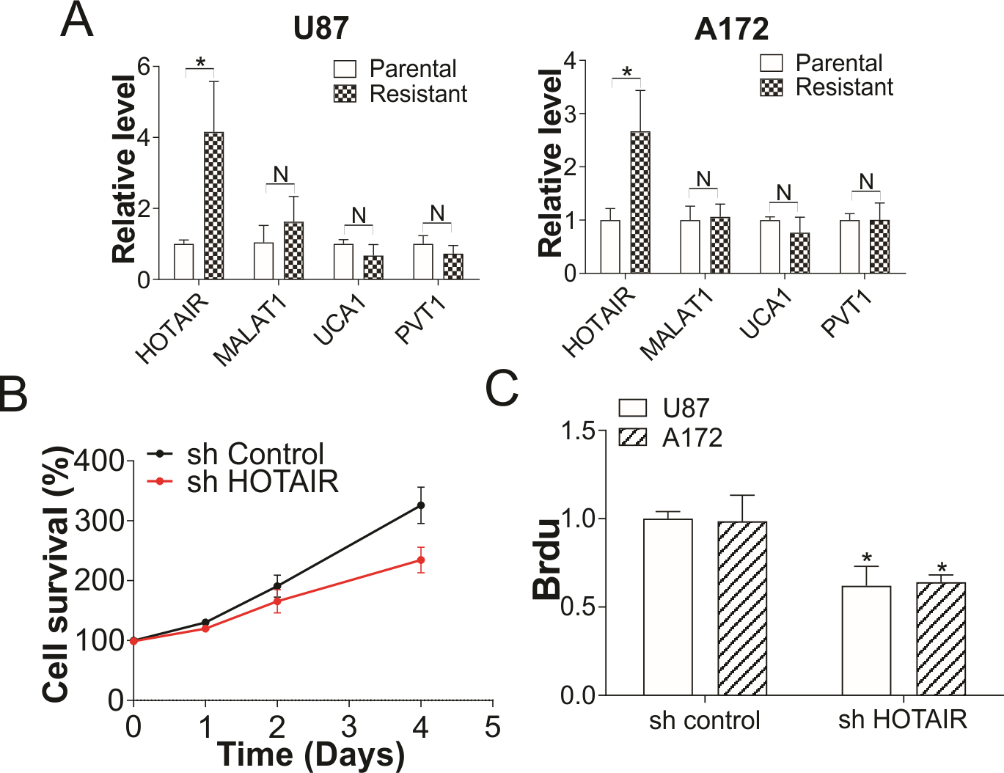


Fig. S3. HOTAIR mediated the GBM cell proliferation. (A) The expression of indicated lncRNA in TMZ parental and resistant U87 (left) and A172 (right) cells. (B) The growth of U87 cells transfected with control or HOTAIR shRNA. (C) The Brdu assay analysis of U87 and A172 cells with control or HOTAIR shRNA transfection. Data were represented in means ± SEM. N, p>0.05; *, p<0.05.


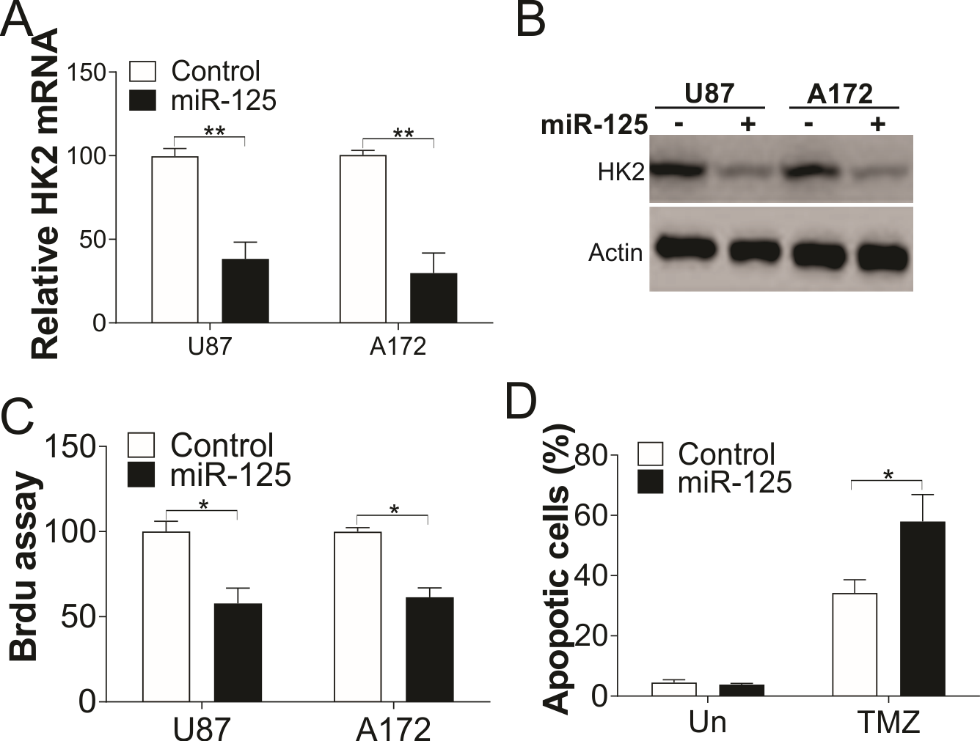


Fig. S4. MiR-125 mediated HK2 expression in GBM cells. (A, B) The mRNA (A) and protein (B) level of HK2 in U87 and A172 cells with control or miR-125 mimic transfection. (C) The Brdu assay of U87 and A172 cells with control or miR-125 mimic transfection. (D) The apoptosis of U87 cells transfected with control or miR-125 mimic and treated with 10 µM TMZ treatment for 24 h. Data were represented in means ± SEM. *, p<0.05; **, p<0.01.
